# Rapamycin treatment increases hippocampal cell viability in an mTOR-independent manner during exposure to hypoxia mimetic, cobalt chloride

**DOI:** 10.1186/s12868-018-0482-4

**Published:** 2018-12-29

**Authors:** Mary A. Zimmerman, Christan D. Biggers, P. Andy Li

**Affiliations:** Department of Pharmaceutical Sciences, Biomanufacturing Research Institute Biotechnology Enterprise (BRITE), North Carolina Central University, Durham, NC USA

**Keywords:** Rapamycin, HT22, Cobalt chloride, Hypoxia

## Abstract

**Background:**

Cobalt chloride (CoCl_2_) induces chemical hypoxia through activation of hypoxia-inducible factor-1 alpha (HIF-1α). Mammalian target of rapamycin (mTOR) is a multifaceted protein capable of regulating cell growth, angiogenesis, metabolism, proliferation, and survival. In this study, we tested the efficacy of a well-known mTOR inhibitor, rapamycin, in reducing oxidative damage and increasing cell viability in the mouse hippocampal cell line, HT22, during a CoCl_2_-simulated hypoxic insult.

**Results:**

CoCl_2_ caused cell death in a dose-dependent manner and increased protein levels of cleaved caspase-9 and caspase-3. Rapamycin increased viability of HT22 cells exposed to CoCl_2_ and reduced activation of caspases-9 and -3. Cells exposed to CoCl_2_ displayed increased reactive oxygen species (ROS) production and hyperpolarization of the mitochondrial membrane, both of which rapamycin successfully blocked. mTOR protein itself, along with its downstream signaling target, phospho-S6 ribosomal protein (pS6), were significantly inhibited with CoCl_2_ and rapamycin addition did not significantly lower expression further. Rapamycin promoted protein expression of Beclin-1 and increased conversion of microtubule-associated protein light chain 3 (LC3)-I into LC3-II, suggesting an increase in autophagy. Pro-apoptotic protein, Bcl-2 associated × (Bax), exhibited a slight, but significant decrease with rapamycin treatment, while its anti-apoptotic counterpart, B cell lymphoma-2 (Bcl-2), was to a similar degree upregulated. Finally, the protein expression ratio of phosphorylated mitogen-activated protein kinase (pMAPK) to its unphosphorylated form (MAPK) was dramatically increased in rapamycin and CoCl_2_ co-treated cells.

**Conclusions:**

Our results indicate that rapamycin confers protection against CoCl_2_-simulated hypoxic insults to neuronal cells. This occurs, as suggested by our results, independent of mTOR modification, and rather through stabilization of the mitochondrial membrane with concomitant decreases in ROS production. Additionally, inhibition of caspase-9 and -3 activation and stimulation of protective autophagy reduces cell death, while a decrease in the Bax/Bcl-2 ratio and an increase in pMAPK promotes cell survival during CoCl_2_ exposure. Together these results demonstrate the therapeutic potential of rapamycin against hypoxic injury and highlight potential pathways mediating the protective effects of rapamycin treatment.

## Introduction

Cobalt chloride (CoCl_2_) induces chemical hypoxia through activation of hypoxia inducible factor-1 alpha (HIF-1α), which is a key pathogenic factor in cerebral ischemia in vivo. As such, CoCl_2_ has been often used as an in vitro hypoxia model, along with oxygen–glucose–deprivation or glutamate exposure, to mimic cerebral ischemia. Cerebral hypoxia, in which the supply of oxygen to the brain is interrupted, is a potent contributor to neurologic dysfunction [1]. Subsequent to an acute lack of oxygen, such as that caused by a stroke or traumatic brain injury (TBI), is the potential for further damage to surviving neuronal tissues due to hyper-activation of inflammatory and various stress or damage-induced cell death pathways, long after the initial insult has been resolved [2–4].

This secondary response, which occurs with reoxygenation, is multifaceted and may involve increases in lipid peroxidation resulting in release of pro-inflammatory second messenger, arachidonic acid [5], along with increases in inflammatory cytokines, such as IL-6, IL-1β and TNFα [6]. Calcium overload, increased glutamate-induced excitotoxicity, perturbations in the mitochondrial membrane and increased oxidative stress have all additionally been associated with both hypoxia and reoxygenation responses [1, 7].

The increase in oxidative stress is particularly detrimental to neurons since these cells rely heavily on mitochondrial oxidative phosphorylation for energy production and, at the same time, have relatively low levels of antioxidants compared to other cells [8]. It should be noted that some ROS generation in the cell is normal and ROS itself has important second messenger functions, however, antioxidizing enzymes, such as glutathione peroxidase and superoxide dismutase, must work to prevent over-accumulation of ROS since they are highly reactive and can cause severe damage to cells when left unchecked. ROS-initiated damage to cell membranes, proteins, lipids, and DNA can result in cell death mechanisms being activated [9, 10].

Such a state of oxidative stress is seen, therefore, when an increase in ROS production occurs that outpaces a cell’s endogenous antioxidant mechanisms. This is often the case during reoxygenation where mitochondria may become overwhelmed when first faced with a burst of oxygen or hypoxia-induced cell damage has compromised mitochondrial detoxification functions [7, 11]. Normal detoxification processes are unable to keep up with the increased production of ROS and the system becomes stressed. Given that, the production of ROS occurs in the mitochondria on the electron transport chain, these organelles become particularly susceptible to ROS-induced damage [8, 11, 12]. For example, ROS has been shown to oxidize mitochondrial proteins, lipids, and DNA and is associated with alterations to the mitochondrial membrane potential (Δψ_m_) [13].

Loss of Δψ_m_, the overall charge difference or electrical gradient across the inner mitochondrial membrane, can severely impact the generation of adenosine triphosphate (ATP) thus affecting a cell’s energetics and increasing susceptibility to death. Disruption of the Δψ_m_ has also been noted in cases of hypoxia and during increased ROS production [13–15]. When oxygen, the final electron accepter in the electron transport chain, is limited during the hypoxic event, electrons flowing through complex IV are hindered and electrons begin to stall in complexes I and III. The lack of electron transfer and concomitant proton pumping across the inner mitochondrial membrane dissipates the Δψ_m_ and prevents coupling to ATP synthase [16]. In addition to this loss of ATP production, extreme changes to the Δψ_m_, for example hyperpolarization as reported with stroke and TBI [15], have been shown to increase ROS production and subsequently trigger formation of the mitochondrial permeability transition pore (mPTP), an opening within the inner mitochondrial membrane that allows for the leakage of ions and other mitochondrial nutrients/proteins. The ensuing changes in concentration gradients along the inner mitochondrial membrane can cause the release of a number of pro-apoptotic components normally sequestered within the intermembrane space.

Such components include the mitochondrial/intrinsic apoptosis pathway initiator proteins, cytochrome C, Smac/Diablo, and apoptosis inducing factor (AIF) [17, 18]. Once released into the cytosol, cytochrome C may combine with pro-caspase-9 and Apaf-1 to form the apoptosome. The apoptosome activates the executioner caspase, caspase-3, a cysteine protease, to induce cell death [19]. Meanwhile, Smac/Diablo works to suppress a group of proteins known as the inhibitors of apoptosis (IAPs) which themselves block caspase activation [17]. AIF migrates to the nucleus where it triggers chromatin condensation and membrane fragmentation [18]. Therefore, damage endured by the mitochondria does not remain confined to these organelles, but rather has important consequences to the overall health of the entire cell.

The initiation of apoptosis can be reduced or enhanced by affecting the level of autophagy that occurs during an insult, such as hypoxia. Autophagy is a catabolic process that breaks down cellular components and nutrients from damaged and dying cells and recycles them potentially to prop up other internal functions or those of neighboring cells. Autophagy has been shown to increase with cerebral hypoxia and other acute brain injuries, but there is still controversy as to whether this increase in autophagy promotes cell survival or instead leads to increased cell death [20, 21]. Its activation can stave off total cell death by replenishing supplies; however, it can also tip the scale towards apoptosis if it becomes over-activated, in which case the cell essentially devours itself. The term mitophagy has been coined to refer to the autophagic process of removing damaged and dysfunctional mitochondria and it has previously been noted that mitophagy is also enhanced with oxidative stress [22, 23].

Rapamycin is a well-documented autophagy activator and is renowned for its ability to decrease mTOR pathway signaling. The mTOR protein can be found in two distinct protein signaling complexes referred to as mTORC1 and mTORC2. In general, mTORC1 responds more directly to environmental stimuli, such as loss of glucose or oxygen, and controls a number of cellular processes, such as protein translation and autophagy, through phosphorylation of downstream kinases, most notably pS6 ribosomal protein, 4E-BP1, and ULK1 [24, 25]. mTORC2 is a recognized effector of insulin signaling and is known to enhance AKT activation [26]. Both complexes respond differently to rapamycin treatment with mTORC1 being more acutely sensitive. mTORC2 typically only becomes disrupted after prolonged, chronic exposure to rapamycin [24]. The interactions between these two pathways, particularly in response to environmental stimuli, is complex and a full description is beyond the scope of this work.

While mTOR activation typically promotes cell survival, in cases of ischemia/reperfusion injury and where oxidative stress is predominant, mTOR signaling may do the opposite as mTOR inhibition has been shown to improve outcomes. For example, rapamycin’s protective effects against hypoxic injury have been documented in the context of cardiac, hepatic, pancreatic, and renal ischemia/reperfusion models [27–32]. The ability of rapamycin to protect against cerebral hypoxic injury has also been reported recently [33–35].

In this study, we used a chemical mimetic of hypoxia, CoCl_2_, to simulate a hypoxic insult in a mouse hippocampal cell line, HT22. CoCl_2_ has been used for this purpose in a variety of cell lines where its primary effect is to increase HIF-1α [36]. HIF-1α stabilization during hypoxia is a widely accepted phenomenon and is central to the activation and regulation of hypoxia response pathways, thus CoCl_2_ exposure mimics hypoxia through modulation of these same response pathways. We have previously noted CoCl_2_’s ability to cause oxidative damage through increased ROS production and hyperpolarization of the mitochondrial membrane in HT22 cells [37]. While not a true measure of hypoxia, we have found it to be a more reliable and consistent model for studying the modifications of hypoxia pathways that are activated similarly during a true hypoxic insult in vivo.

Our objective was to determine if treatment with rapamycin could confer protection against CoCl_2_-simulated hypoxic injury. Specifically, we looked to determine if rapamycin could mitigate changes in cell viability, mitochondrial membrane potential, and ROS production. Additionally, we sought to identify key pathways that may contribute to rapamycin protection against hypoxia.

## Results

### Exposure to CoCl_2_ significantly reduces HT22 cell viability, while treatment with rapamycin increases viability during exposure

**Fig. 1:**
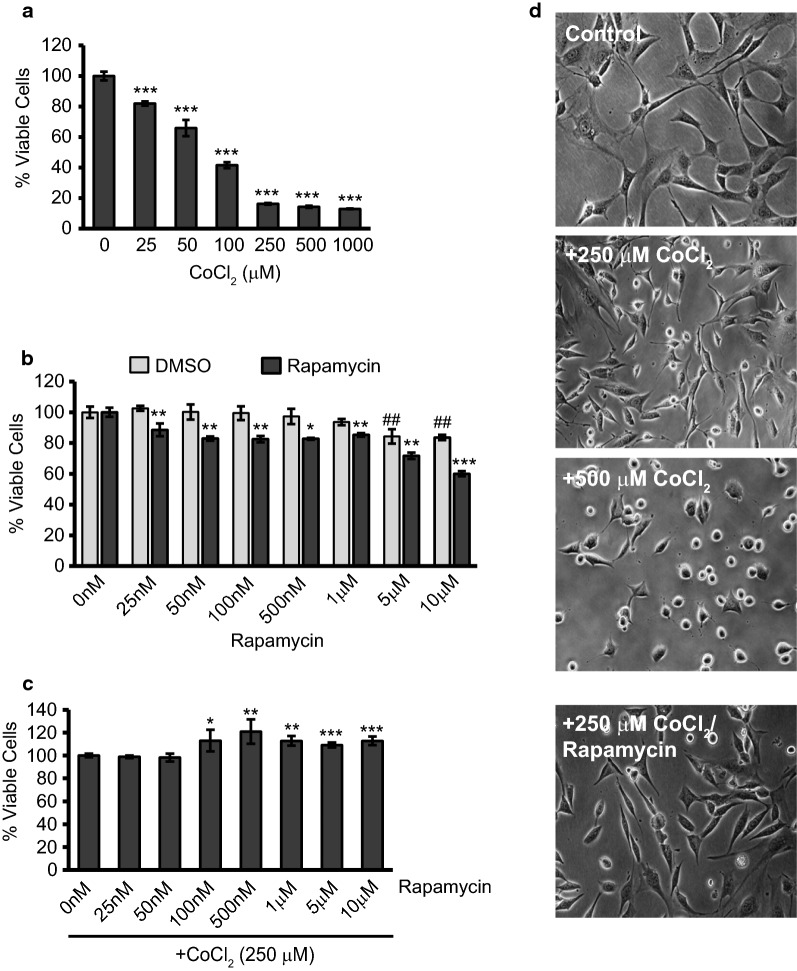
Exposure to CoCl_2_ significantly reduces viability, while treatment with rapamycin increases viability during exposure. **a** CoCl_2_ decreases HT22 viability in a dose-dependent manner. HT22 cells were treated at 70% confluence in 96-well plates with the indicated concentrations of CoCl_2_ (0–1 mM) for 24 h followed by assessment of cell viability using the resazurin viability assay. ***p < 0.001 versus control. **b** Assessment of changes in cell viability caused by rapamycin treatment only. Cells were treated with the indicated amount of rapamycin (0-10 µM), or an equal volume containing the same percent of DMSO, for 24 h before assessing viability as in A. ^##^p < 0.01 versus control; *p < 0.05 versus matched DMSO; **p < 0.01 versus matched DMSO; ***p < 0.001 versus matched DMSO. **c** Rapamycin-induced protection during CoCl_2_ exposure. HT22 cells were exposed to 250 µM CoCl_2_ for 24 h together with the indicated doses of rapamycin. Assessment of viability was performed with the resazurin viability assay. *p < 0.05 versus control; **p < 0.01 versus control; ***p < 0.001 versus control. **d** Morphological assessment after CoCl_2_ exposure and rapamycin treatment. Representative images are shown from cells exposed 24 h to 0, 250, or 500 µM CoCl_2_. An additional group was exposed for 24 h with 250 µM CoCl_2_ plus 500 nM rapamycin. Photomicrographs were taken under a standard light microscope at 20X magnification

Before we could examine the potential protection with rapamycin treatment, we tested a range of CoCl_2_ doses to establish a hypoxia model in HT22 cells. HT22 cells were exposed for 24 h with CoCl_2_ only, ranging from 25 µM to 1 mM (Fig. 1a). Assessment of resultant cell viability was through the resazurin blue assay which revealed a dose-dependent response causing significant decreases in viability at all doses tested (p ≤ 0.001). We chose 250 µM CoCl_2_ to be used in our subsequent experiments as this produced a 75% decrease in cell viability and caused pronounced changes in cell morphology as seen in Fig. 1d. With 250 and 500 µM CoCl_2_ exposure, cells elongate, taking on a more flattened appearance, before rounding and detaching from the culture flask as they die off from CoCl_2_ exposure. The sensitivity of HT22 cells to CoCl_2_ exposure is in line with our previously published report showing CoCl_2_-induced viability loss [37] and a report by Yang et al. [38] which showed roughly 50% loss in HT22 viability after 16 h exposure to 500 µM CoCl_2_.

We also determined the effects on viability of rapamycin itself using a range of 25 nM to 10 µM. Because rapamycin is dissolved in dimethyl sulfoxide (DMSO) we analyzed our results against DMSO groups containing equal volumes and percent of DMSO. Rapamycin treatment itself had a detrimental effect on HT22 cells averaging a 15% decrease in viability when compared to DMSO-induced viability losses (Fig. 1b). DMSO only became significantly toxic at the two highest doses tested (p ≤ 0.01) and, even then, rapamycin further decreased viability in comparison by 24% (p ≤ 0.001).

Despite these findings, we tested CoCl_2_-exposed cells together with this same range of rapamycin concentrations. As can be seen in Fig. 1c, starting at 100 nM, rapamycin addition caused significant gains in cell viability culminating in a 21% increase with 500 nM rapamycin treatment (p ≤ 0.01). Even at the highest doses of rapamycin, which from Fig. 1b we knew DMSO would begin to effect viability, we still obtained increases in viability of around 10% (p ≤ 0.001). Since it yielded the greatest increase in viability against CoCl_2_ without having a DMSO effect and was additionally able to reduce the morphological effects attributed to CoCl_2_ exposure (Fig. 1d), we choose the 500 nM rapamycin concentration to use in all subsequent experiments.

### Rapamycin treatment limits caspase-9 and caspase-3 activation to promote cell survival

**Fig. 2:**
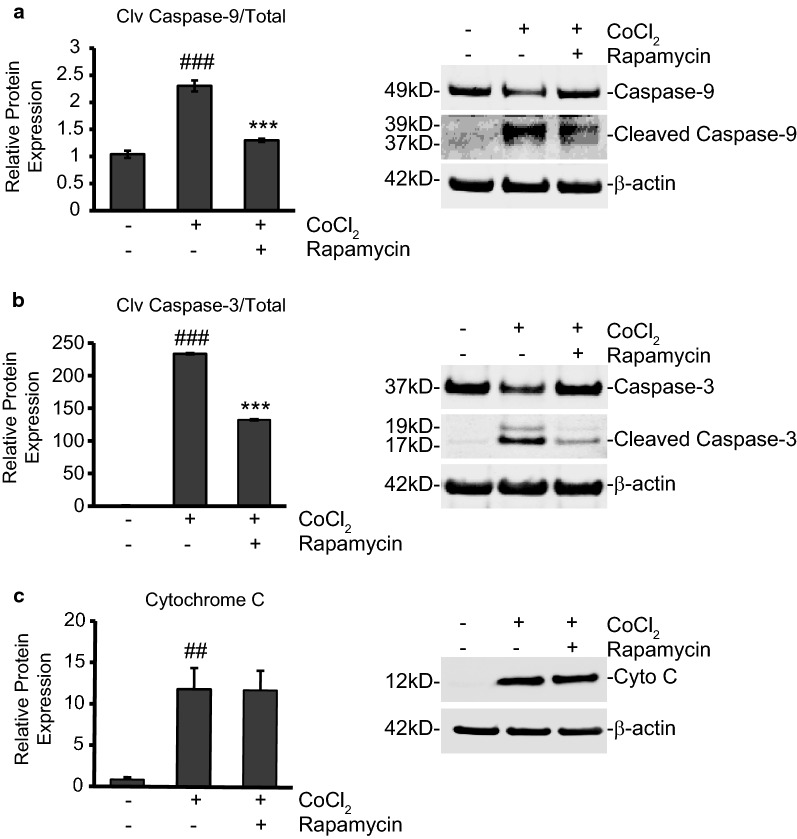
Rapamycin treatment limits caspase-3 activation to promote cell survival during CoCl_2_ exposure. **a** Rapamycin treatment reduces initiator caspase-9 activation. HT22 cells were untreated or treated with 250 µM CoCl_2_, with and without 500 nM rapamycin for 24 h. Cells were lysed to obtain cytosolic protein fractions and cleaved caspase-9 protein expression was analyzed by western blot. Left is the average relative protein expression of each group when normalized to total caspase-9 protein expression. Right is a representative image of at least three separate blots showing caspase-9 protein expression. ^###^p < 0.001 versus control; ***p < 0.001 versus CoCl_2_ alone. **b** Rapamycin treatment reduces executioner caspase-3 activation. Cells were treated, and cytosolic protein fractions obtained and analyzed as in A. Left is the average relative protein expression of cleaved caspase-3 when normalized to total caspase-3 protein expression. Right is a representative image of caspase-3 protein expression from at least three separate blots. ^###^p < 0.001 versus control; ***p < 0.001 versus CoCl_2_ alone. **c** Rapamycin does not affect CoCl_2_-induced cytochrome C release. Cells were treated, and cytosolic protein fractions obtained and analyzed as in A. Left is the average relative protein expression of cytochrome C when normalized to β-actin protein expression. Right is a representative image of cytochrome C protein expression from at least three separate blots. ^##^p < 0.01 versus control

Caspase-3 plays a pivotal role in causing apoptosis in neurons following ischemia [39]. Once cleaved by caspase-9, the activated caspase-3 proceeds to cause chromatin condensation and nuclear fragmentation resulting in apoptosis of the cell. We used Western blot analysis to measure changes in protein expression of this key marker of apoptosis, along with expression of its initiator, caspase-9. The overall ratio of cleaved caspase-9 to its uncleaved form increased by 121% (p < 0.001) during CoCl_2_ exposure and was reduced from this high by 43% when rapamycin was added (p < 0.001; Fig. 2a). More significantly, 24 h exposure to CoCl_2_ caused a roughly 140-fold increase in the ratio of cleaved caspase-3 compared to its uncleaved form (p ≤ 0.001) evidencing the high level of apoptosis induced by CoCl_2_. Rapamycin treatment reduced this activation by roughly 40% (p ≤ 0.01; Fig. 2b). While caspase-3 activation can occur through activation of the extrinsic cell death pathway (engagement of the death receptors, Fas, TRAIL, etc.), the intrinsic cell death pathway, initiated through mitochondrial damage and release of cytochrome C into the cytosol, also activates caspase-3. Indeed, cytochrome C expression was highly upregulated, more than 12-fold, in the cytosol protein fraction after CoCl_2_ exposure (Fig. 2c; p ≤ 0.01), illustrating the damaging effect of CoCl_2_ exposure on mitochondrial membrane integrity. However, despite being able to limit caspase-9 and -3 activation, rapamycin addition was unable to prevent the release of cytochrome C from mitochondria.

### Rapamycin treatment restores the Δψ_m_ and decreases the production of ROS in CoCl_2_-exposed cells to limit hypoxia-induced damage

**Fig. 3:**
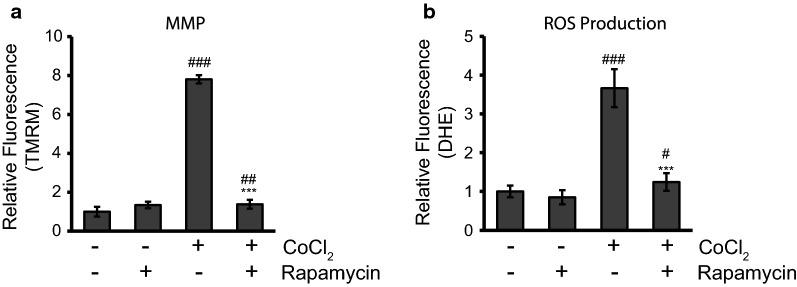
Rapamycin treatment restores the Δψ_m_ and decreases the production of ROS in CoCl_2_-exposed cells. **a** Rapamycin treatment prevents hyperpolarization of the mitochondrial membrane. HT22 cells were untreated or treated with 250 µM CoCl_2_ with and without 500 nM rapamycin for 24 h. 500 nM TMRM dye was added during the final 30 min of treatment. Cells were washed with PBS and fluorescence was measured using a PHERAstar Microplate Reader with a 590-50/675-50 filter. Relative fluorescence intensities were obtained by subtracting background signal from treated cells without TMRM/DHE and normalizing to the percent of viable cells. ^##^p < 0.01 versus control; ^###^p < 0.001 versus control; ***p < 0.001 versus CoCl_2_ alone. **b** Rapamycin treatment decreases ROS production. Cells were treated and analyzed as in A except 5 µM DHE dye was added instead of TMRM. ^#^p < 0.05 versus control; ^###^p < 0.001 versus control; ***p < 0.001 versus CoCl_2_ alone

Having noted the large increase in cytochrome C release caused by CoCl_2_ it was clear the mitochondria were being targeted in our hypoxia model. We measured Δψ_m_ using a fluorogenic dye, tetramethylrhodamine, methyl ester (TMRM), as described in the materials and method (Fig. 3a). Rapamycin by itself caused no statistically significant change to the Δψ_m_ and averaged an increase in signal of only 34%. On the other hand, CoCl_2_ exposure for 24 h significantly upregulated the TMRM fluorescent signal, increasing it by 680% (p ≤ 0.001). This nearly sevenfold increase indicated severe hyperpolarization of the mitochondrial membrane. When rapamycin was added together with CoCl_2_, this hyperpolarization was markedly inhibited (82% reduction) compared to CoCl_2_ alone (p ≤ 0.001) and fluorescence remained only slightly elevated when compared to the control (38%; p ≤ 0.01).

As noted earlier hyperpolarization of the mitochondrial membrane has been linked to increased ROS production. That, and because of the large body of evidence showing hypoxic injury stems from increased ROS production, we examined rapamycin’s ability to modify ROS production in our model. ROS production was measured using another fluorogenic dye, dihydroethidium (DHE), as described in the materials and method. CoCl_2_ caused a nearly threefold increase in ROS production; an increase of 266% (p ≤ 0.001) (Fig. 3b). Rapamycin by itself had no statistically significant effect on ROS but did cause average ROS production to drop by 15%. Rapamycin added together with CoCl_2_, significantly reduced ROS production by 66% when compared to CoCl_2_ alone (p ≤ 0.001) and ROS production remained only slightly elevated above untreated control levels (24%; p ≤ 0.05).

### Rapamycin treatment effects during CoCl_2_ exposure are independent of changes in mTOR, but can interfere with autophagy

**Fig. 4:**
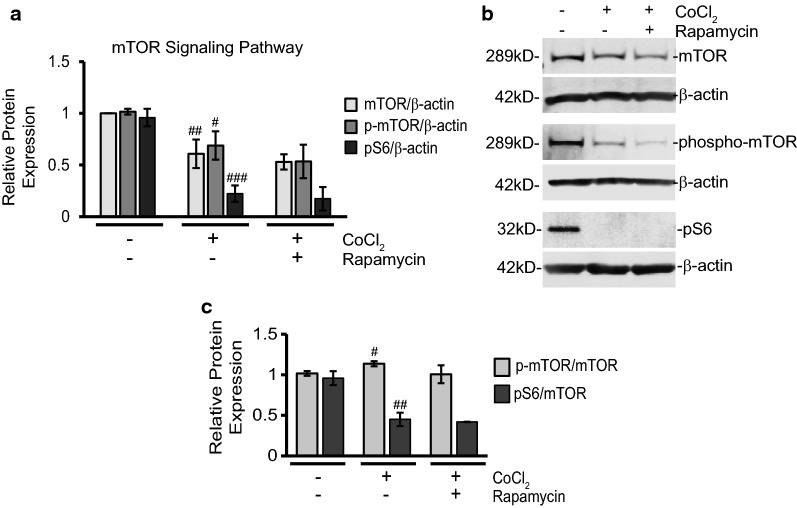
CoCl_2_ exposure reduces mTOR signaling while rapamycin is unable to further alter pathway expression profile. **a** Rapamycin treatment does not restore mTOR protein expression profile during CoCl_2_ exposure. Cells were treated and analyzed as in Fig. 2a via Western blot. Shown is the average relative protein expression of mTOR, p-mTOR, and pS6 in each group when normalized to β-actin protein expression. ^#^p < 0.05 versus control; ^##^p < 0.01 versus control; ^###^p < 0.001 versus control. **b** Representative images of at least three separate blots (cells treated as in A) showing expression of the indicated mTOR signaling proteins along with loading control β-actin. **c** Ratio of p-mTOR and pS6 to total mTOR levels. Cells were treated and analyzed as in A via Western blot. Shown are the average ratios of p-mTOR and pS6 in each group to total mTOR levels. ^#^p < 0.05 versus control; ^##^p < 0.01 versus control

After observing the detrimental effects of CoCl_2_ exposure and determining that rapamycin could subvert these changes, we began exploring the molecular pathways mediating these responses. We first examined the traditional signaling pathways rapamycin is known to affect, namely mTOR and autophagy. Figure 4 shows our Western blot analysis of mTOR and phosphorylated mTOR (p-mTOR) protein expression following exposure to CoCl_2_, both with and without rapamycin addition. Both mTOR and p-mTOR showed significant down regulation after CoCl_2_ exposure alone when normalized to β-actin (39 and 32% respectively; p ≤ 0.01; Fig. 4a). The overall ratio of p-mTOR to mTOR was slightly elevated after CoCl_2_ exposure (p < 0.05), but not with rapamycin treatment (Fig. 4c). While rapamycin treatment appeared to further decrease mTOR and p-mTOR expression (13 and 23%) from CoCl_2_ alone, the differences were not statistically significant. Expression of pS6 is often used as a downstream readout for mTOR signaling. We also found pS6 protein expression to be severely inhibited (77%; p ≤ 0.001;) by CoCl_2_ when normalized to β-actin (Fig. 4a) and when analyzed as a ratio of total mTOR (53% reduction; p < 0.01; Fig. 4c). Addition of rapamycin, like with mTOR and p-mTOR, was unable to reverse this effect.

### Rapamycin treatment enhances autophagy to protect cells during CoCl_2_ exposure

**Fig. 5:**
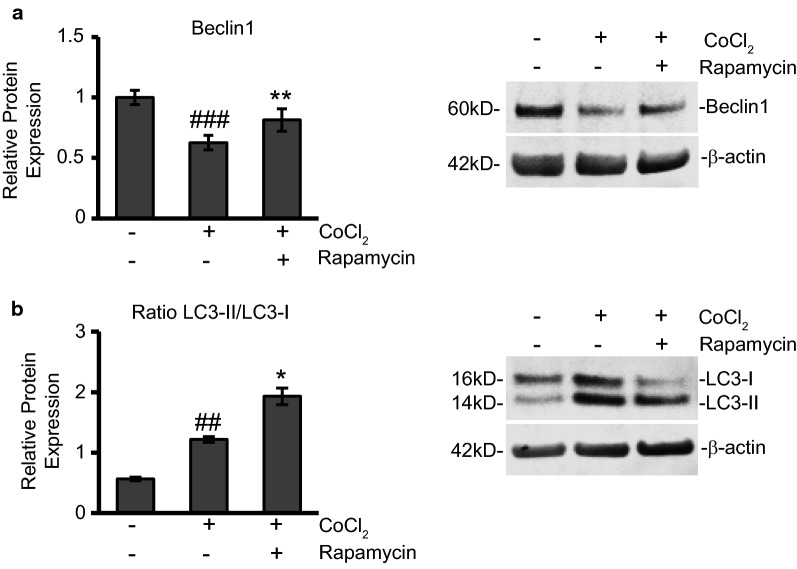
Rapamycin treatment favors autophagy during CoCl_2_ exposure. **a** Rapamycin treatment restores expression of autophagy marker, Beclin1. Cells were treated and analyzed as in Fig. 5a via Western blot. Left is the average relative protein expression of Beclin1 normalized to β-actin protein expression. Right is a representative image of at least three separate blots showing Beclin1 protein expression. ^###^p < 0.001 versus control; **p < 0.05 versus CoCl_2_ alone. **b** Ratio of LC3-II/LC3-I is elevated during CoCl_2_ exposure and further increased with rapamycin addition. HT22 cells were treated as in Fig. 2a. Cells were lysed to obtain total protein fractions and the ratio of LC3-II to LC3-I protein expression was analyzed by Western blot. Left is the average relative ratio of LC3-II/LC3-I protein expression of each group. Right is a representative image of at least three separate blots showing LC3-I and LC3-II protein expression. ^##^p < 0.01 versus control; *p < 0.05 versus CoCl_2_ alone

The other well-documented effect of rapamycin therapy is the activation of autophagy. As mentioned previously, the role of autophagy, as it relates to improving outcome or being deleterious when faced with an insult, is unclear. In our present study we used Western blot to measure protein expression changes in the autophagy marker, Beclin-1 (Fig. 5a). Beclin-1 is involved in the early stages of autophagy where it plays a role in localizing autophagic proteins to a pre-autophagosomal structure [40]. We found in our model that Beclin-1 expression was reduced by 37% during CoCl_2_ exposure (p ≤ 0.001). Rapamycin treatment increased Beclin-1 by 30% (p ≤ 0.01) restoring levels close to control expression.

To further confirm autophagy induction we used Western blot to measure protein expression of LC3-I and LC3-II. Autophagy flux was determined by calculating the ratio of LC3-II/LC3-I in each experimental condition (Fig. 5b). Conversion of LC3-I to LC3-II has been correlated to autophagosome formation that occurs during autophagy and therefore the amount of LC3-II/LC3-I can be compared between samples as an indicator of autophagy induction [41]. We observed that untreated cells had low levels of autophagy induction as the ratio of LC3-II/LC3-I was around 0.5. With CoCl_2_ exposure LC3-II to LC3-I expression was observed at a 1:1 ratio (p ≤ 0.0.1). Finally, when rapamycin was added during CoCl_2_ exposure, the LC3-II/LC3-I ratio increased to 2 (p ≤ 0.05).

### Rapamycin treatment reduces the Bax/Bcl-2 ratio and increases pMAPK protein expression to promote survival during CoCl_2_ exposure

At this point, we concluded rapamycin might be exerting its pro-survival influence through mTOR-independent mechanisms. Besides the downstream inhibition of caspase-3 activation and an increase in autophagy, the pathways responsible for mediating the protective effects of rapamycin were unclear. Since mTOR signaling was not significantly altered with treatment, we focused on key survival mediators involved in mitochondrial damage responses that could be linked to ROS production. The Bcl-2 protein family is a key regulator of the mitochondrial apoptosis pathway and contains members responsible for both pro- and anti-apoptotic responses. An imbalance in the ratio of these pro- and anti-apoptotic proteins can determine a cell’s fate by either promoting or hindering mitochondria-initiated apoptosis. In addition to mediating the intrinsic apoptosis pathway, which is of particular interest to our study, these members have also been shown to be involved in oxidative stress-induced neuronal injury [42].

**Fig. 6:**
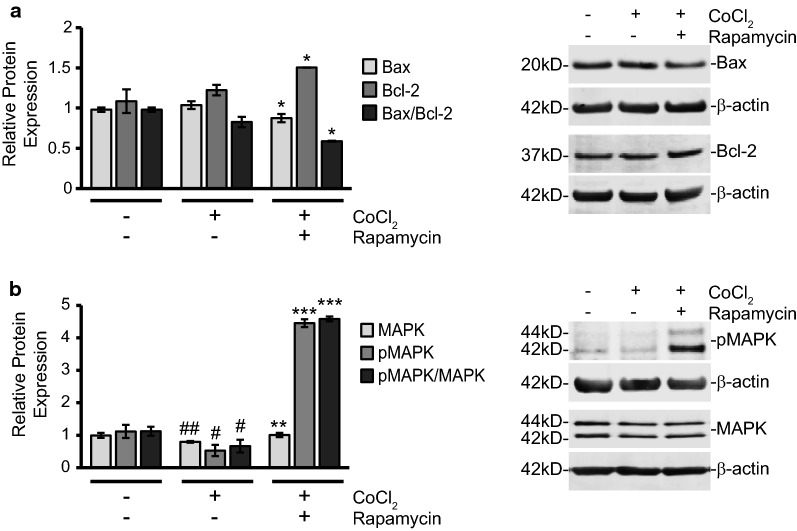
Rapamycin treatment upregulates Bcl-2 and pMAPK to promote survival during CoCl_2_ exposure. **a** Rapamycin treatment increases expression of anti-apoptotic Bcl-2 protein while reducing pro-apoptotic Bax expression. Cells were treated and analyzed as in Fig. 5a via Western blot. Left are the average relative protein expressions of Bax, and Bcl-2 in each group when normalized to β-actin protein expression. The average ratio of Bax to Bcl-2 expression was also calculated and is shown. Right is a representative image of at least three separate blots showing Bax and Bcl-2 protein expression. *p < 0.05. **b** The ratio of phosphorylated to unphosphorylated MAPK is increased with rapamycin treatment during CoCl_2_ exposure. Cells were treated and analyzed as in Fig. 2a via Western blot. Left are the average relative protein expressions of MAPK and pMAPK normalized to β-actin protein expression. Ratio was obtained by dividing relative protein expression values of pMAPK by the relative protein expression values of unphosphorylated MAPK. Right is a representative image of at least three separate blots showing MAPK and pMAPK protein expression. ^#^p < 0.05 versus control; ^##^p < 0.01 versus control; **p < 0.01 versus CoCl_2_ alone; ***p < 0.001 versus CoCl_2_ alone

The family’s namesake protein, Bcl-2, is primarily an anti-apoptotic protein whose increase is deemed beneficial for cell survival. Using Western blot analysis, we measured changes in Bcl-2 protein expression (Fig. 6a). With rapamycin treatment added during CoCl_2_ exposure, we observed a 38% increase in Bcl-2 protein expression when compared to control cells and a 23% increase when compared to CoCl_2_ alone (p ≤ 0.05). CoCl_2_ alone caused a 12% increase in Bcl-2 but the difference was not statistically significant.

Another member of the Bcl-2 protein family, with strong ties to the intrinsic cell death pathway, is the apoptosis-promoter, Bax. Bax is often noted to form a complex with Bcl-2 and the Bax/Bcl-2 ratio often acts as a rheostat for cell survival versus apoptosis induction [43]. A low Bax/Bcl-2 ratio is typically seen as favoring cell survival while a high Bax/Bcl-2 ratio will often tip cells towards cytochrome C release and mitochondria-initiated apoptosis. After treatment with CoCl_2_, we noted that Bax protein expression was increased 6%, but the difference was not statistically significant. However when compared to CoCl_2_ alone, rapamycin addition significantly decreased Bax expression by 16% (p ≤ 0.05). The changes in Bax expression were nearly equal and opposite to those of Bcl-2. During CoCl_2_ exposure, the overall Bax/Bcl-2 ratio was significantly decreased (29%; p ≤ 0.05) with rapamycin treatment (Fig. 6a).

After observing the amplification of Bcl-2 and decrease in Bax expression, we measured changes in both unphosphorylated and phosphorylated 44/42 MAPK (pMAPK) proteins. This intracellular signaling molecule is a well-documented promoter of cell survival, particularly in neurons, and has pronounced effects on apoptosis, cell proliferation, growth and differentiation [44, 45]. Furthermore, the activation of MAPK through phosphorylation has also been related to detoxification of ROS [46–48] and we hypothesized it could play a role in our CoCl_2_-simulated hypoxia model. As seen in Fig. 6b both MAPK and pMAPK protein expression were significantly reduced by 20 and 85% respectively (p ≤ 0.01 and p ≤ 0.05) in CoCl_2_-exposed cells. Rapamycin addition increased expression of MAPK by 26% (p ≤ 0.05) and pMAPK by 740% (p ≤ 0.001) when compared to CoCl_2_ exposed cells. After normalizing both MAPK and pMAPK to β-actin expression, we calculated the ratio of pMAPK to MAPK expression. As seen in Fig. 6b, CoCl_2_ treatment alone significantly reduced the pMAPK/MAPK expression ratio by 41% (p ≤ 0.01). Rapamycin during CoCl_2_ exposure not only restored the expression ratio of pMAPK/MAPK, but dramatically upregulated its expression nearly sixfold (p ≤ 0.001). pMAPK/MAPK expression was increased 309% from control levels and 592% from CoCl_2_ exposure alone.

## Discussion

Using CoCl_2_ to mimic a hypoxic insult to hippocampal cells, we tested the therapeutic efficacy of rapamycin, a natural compound produced by bacteria and known to have antibiotic, antifungal, and immunosuppressant functions. With the addition of rapamycin during CoCl_2_ exposure, we observed increased cell viabilities, reduced caspase-9 and caspase-3 activation, stabilization of the Δψ_m_, and significant reduction in ROS production. We believe protection with rapamycin treatment may be conferred independent of changes in mTOR signaling itself, but rather through increased autophagy, a decrease in the Bax/Bcl-2 ratio and increased MAPK activation.

Previous studies examining rapamycin’s ability to limit hypoxia-induced damage, the bulk of which have occurred in the context of cardiac ischemia and reperfusion, have noted overall increases in cell survival [27–32]. The protective mechanisms most attributed to these increases in cell survival were changes in autophagy and mTOR signaling. However, within these studies, mTOR has been found to have both cardioprotective and cardiotoxic effects complicating the precise role of mTOR in mediating ischemic damage [49, 50]. While we too found rapamycin promoted cell survival in hypoxic neurons, our results indicate a less prominent role for the mTOR signaling pathway. However, it should be noted that CoCl_2_ exposure, by itself, down-regulated the mTOR signaling pathway as evidenced by significant decreases in mTOR, p-mTOR, and pS6 protein expression (Fig. 4).

This is not surprising, as mTOR has been reported to decrease in the brain during stressful situations, such as glucose deprivation, DNA damage, and hypoxia [51]. However, as with mTOR in cardiac ischemia, there is still uncertainty as to the role of mTOR in mediating cerebral stroke damage in vivo. In our previous studies, we have utilized a transient global cerebral ischemia model in rats to examine the correlations between mTOR signaling and neuronal cell death during fixed reperfusion intervals. At these intervals, we noted marked increases in mTOR pathway components that corresponded to greater cell death. Rapamycin was shown in these cases to reduce mTOR singaling as well as prevent cell death [33–35].

In our present study, we did not examine samples from a reperfusion or reoxygenation stage, but instead collected neuronal cells while they were still in a hypoxia-simulated environment. A limitation of using a cell line for these experiments is a loss of any whole-animal in vivo effects. The use of HT22 cells, however, allowed us to conduct molecular-based experiments that would otherwise be cumbersome in an animal model. In our analysis we found that, in addition to decreased mTOR expression, cell death was also more pronounced under the CoCl_2_ exposure condition, with a nearly 80% decrease in cell viability (Fig. 1) and a more than 140-fold increase in cleaved caspase-3 protein expression (Fig. 2). Despite the contrast in mTOR expression levels seen in these two models, rapamycin still conferred protection in both cases. In this regard, the protective actions of rapamycin against CoCl_2_-simulated hypoxia may be different in that they do not stem from inhibiting an increase in mTOR expression as occurs during reperfusion in an in vivo stroke model. Nor does the protective benefit arise, as our results in Fig. 4 indicate, from any significant reduction in mTOR expression with rapamycin treatment. Beyond the scope of our study was ascertaining the degree to which this depression in mTOR signaling was directly responsible for reducing cell viability during CoCl_2_ exposure. What we did find, was that rapamycin treatment did not further reduce mTOR expression from the already low levels induced by CoCl_2_ exposure, nor did treatment in any way reverse this depression. This leads us to conclude that rapamycin protection against CoCl_2_ is independent of mTOR signaling.

Based on our results, we believe the induction of autophagy, which is often a result of inhibited protein translation/amino acid deprivation following mTOR inhibition, plays a more central role in improving viability during CoCl_2_ exposure. We know that cells exposed to CoCl_2_ undergo a great deal of stress. There is a considerable increase in toxic ROS production (Fig. 3b) and the mitochondrial membrane potential is substantially altered (Fig. 3a). Autophagy may serve in this context to remove damaged/dying mitochondria and provide recycled components to help prop up remaining healthy mitochondria in an effort to alleviate oxidative stress. This would explain how rapamycin, an activator of autophagy, was able to decrease ROS production and stabilize the mitochondrial membrane. Evidence of autophagy, specifically autophagy upregulated by rapamycin treatment, conferring protection against cardiac and neurological insults exists in the literature [52–54]. We saw too in our results that CoCl_2_-induced reduction of mTOR expression also yielded a significant increase in the LC3-II/LC3-I ratio (Fig. 5a) however this did not produce a survival benefit as CoCl_2_ exposure increased caspase-3 activation 140-fold and cytochrome c release tenfold (Fig. 2a, b). CoCl_2_ exposure also, in contrast to the increased LC3-II/LC3-I ratio, reduced the early autophagy mediator, Beclin-1 (Fig. 5b). It was only when rapamycin treatment was added that Beclin-1 expression was restored and the LC3-II/LC3-II ratio again doubled. With this rapamycin-induced increase in autophagy activation, we noted a roughly 20% increase in cell viability (Fig. 1c).

Autophagy may be just one mechanism for rapamycin-induced protection. In addition to the increase in autophagy, we noted an increase in anti-apoptotic Bcl-2 and a simultaneous decrease in pro-apoptotic Bax with rapamycin treatment. Both of these proteins can mediate the mitochondrial intrinsic death pathway by facilitating mitochondrial outer membrane permeability (MOMP) and the release of cytochrome c. Bax is able to insert itself into the outer mitochondrial membrane to trigger MOMP while binding with Bcl-2, also located on the outer mitochondrial membrane, serves to deactivate Bax [42].

Bcl-2 has long been shown to confer protection against a variety of neurological insults including oxidative stress, excitotoxicity, and cerebral ischemia [42]. Mice deficient in Bcl-2 also experience enhanced oxidative stress and alterations in antioxidants in the brain [55]. It is possible the increase in Bcl-2 expression we saw during rapamycin treatment also contributed to the observed decrease in ROS production with rapamycin addition (Fig. 3b) although we did not measure this connection directly. In opposition to Bcl-2, which confers protection, increased Bax has previously been associated with oxidative damage in neurological models [42]. Bax and Bcl-2 proteins often serve as counterbalances to one another and, with rapamycin addition, the ratio of Bax to Bcl-2 during CoCl_2_ exposure significantly dropped (Fig. 6a) favoring cell survival. Even though we did not detect significant prevention of cytochrome c release with rapamycin addition, caspase-9 and caspase-3 activation were significantly reduced (Fig. 2a, b) suggesting CoCl_2_-induced caspase-3 cleavage is independent of cytochrome C release.

Caspases are known to play a pivotal role in ischemia-induced apoptosis. While necrotic cell death predominates in the primary injury location, the surrounding areas often experience apoptosis as a delayed response [56]. This delay allows a therapeutic window in which interventions can be made to prevent the induction of apoptosis in these adjoining cells in hopes of minimizing the overall damage to the patient. Most of this apoptosis during the secondary response is triggered by an increase in ROS production [16]. Interestingly, activation of MAPKs has been related to detoxification of ROS and protection against oxidative stress [57]. Upregulation of MAPK has also been linked to increased Bcl-2 expression on human neuron-like cells [58]. Both of these findings are supported by this present work. We found that both MAPK and pMAPK were significantly reduced during CoCl_2_ exposure (Fig. 6b). With rapamycin treatment, in which we see protection, pMAPK is significantly upregulated, as well as the total pMAPK/MAPK ratio. Activation of MAPK may also explain why we witnessed a decrease in cleaved caspase-9 and caspase-3 expression, but not cytochrome C release (Fig. 2a, b). MAPK has previously been shown to function downstream of cytochrome c release [59] and has been shown to interfere specifically with caspase-9 activation [60]. This would explain a reduction in cleaved caspase-9 and caspase-3 expression with rapamycin treatment. MAPK signaling is certainly diverse, and its functions can differ depending on the cell type and context [61], however, this pro-survival function is in line with the vast majority of studies describing pMAPK signaling in neurons [44]. It is also in line with increased MAPK signaling acting as a protective mechanism against hypoxic insults [57, 62, 63].

## Conclusions

In summary, our research indicates that rapamycin confers protection during CoCl_2_-simulated hypoxia by several mechanisms. At the forefront of these mechanisms are an increase in autophagy and concomitant decrease in apoptosis. While the role of rapamycin acting on autophagy is widely recognized, the action on caspases is more novel and delineation of this response requires further study. Rapamycin-treated cells also experience a reduction in ROS production and restoration of the mitochondrial membrane, two findings highly relevant towards achieving protection against hypoxia. The molecular mechanisms contributing to these gross responses are a decrease in the Bax/Bcl-2 ratio and an increase in pMAPK. Taken together, our results highlight potential pathways outside of mTOR responsible for rapamycin’s protective effects and give credence to rapamycin as a promising agent against neuronal hypoxia.

## Methods

### Cell culture

HT22 is a mouse hippocampal cell line kindly provided by Dr. Jun Panee at the University of Hawaii. HT22 cells were cultured in Dulbecco’s Modified Eagles Medium–High Glucose (GE Healthcare Life Sciences, Marlborough, MA) supplemented with 10% Fetal Bovine Serum, l-glutamine (2 mM), and antibiotics penicillin G and streptomycin (200 units/mL) (Thermo Fisher Scientific, Waltham, MA). Cells were maintained in a % 5 CO_2_ incubator at 37 °C, and 90–95% humidity. Cells were harvested using a 0.05% trypsin solution (Lonza Bioscience, Walkersville, MD).

### Measurement of cell viability via resazurin assay

Cell viability was measured using a water-soluble, indicator dye, resazurin (7-Hydroxy-3H-phenoxazin-3-one 10-oxide), essentially as previously described [31]. Resazurin sodium salt (Acros Organics, Morris Plains, NJ) was dissolved in media at a final concentration of 0.1 mg/mL and 10% volume. This cell-permeable dye is internalized by cultured cells. Actively growing cells metabolize resazurin into a fluorescent form, resorufin. Resultant fluorescence was measured using a PHERAstar Microplate Reader (BMG Labtech, Cary, NC) with a 540-20/590-20 filter. The viability of control cells was arbitrarily set to 100% and the relative fluorescence intensities of experimental groups were converted to relative percentages using the formula: (Relative Fluorescence Intensity of Experimental/Average Relative Fluorescence Intensity of Control) × 100 = % of viable cells.

### Western blotting

Cells for western blot analysis were lysed to obtain cytosolic and mitochondrial protein fractions. To obtain these fractions, cells were first lysed in cytosol extraction buffer (250 mM sucrose, 70 mM KCl, 137 mM NaCl, 4.3 mM Na_2_HPO4, 1.4 mM KH_2_PO4 pH 7.2, and 200 µg/mL digitonin) plus phosphatase and proteinase inhibitors (Pierce and Thermo Fisher Scientific, Waltham, MA) on ice for 5 min. Cells were centrifuged at 1000×*g* for 5 min at 4 °C, reserving the supernatant as the cytosolic fraction. The cytosolic fraction was further cleared of debris by centrifugation at 20,000×*g* for 10 min at 4 °C. Meanwhile, the mitochondrial fractions were obtained by incubating the pellet from the first, low-speed centrifugation in two volumes of mitochondrial lysis buffer (50 mM Tris–HCl pH 7.4, 150 mM NaCl, 2 mM EDTA, 2 mM EGTA, 0.2% (v/v) Triton X-100, and 0.3% NP-40) plus the above inhibitors.

Where indicated, total cell protein lysates were used for Western blots. To obtain these lysates, cells were incubated on ice for 30 min in RIPA Buffer Solution (Teknova, Hollister, CA) supplemented with the same inhibitors used for cytosolic and mitochondrial fractions. Cells were centrifuged at high speed for 20 min and protein concentrations were measured from the resulting supernatants using standard Bradford Assays (Bio-Rad Laboratories, Hercules, CA).

Protein lysates (20 µg per well) were separated using 4–12% Bis–Tris NuPAGE gels except in the cases of mTOR/phosho-mTOR detection where 3–8% Tris–Acetate NuPAGE gels were used according to the manufacturer’s instructions (Invitrogen, Carlsbad, CA). The Bio-Rad Mini Trans-Blot system was used to transfer the separated proteins to PVDF membranes. After transfer, membranes were blocked in a 1:1 solution of Li-COR Odyssey Blocking buffer (Li-COR, Inc., Lincoln, NE) and PBS. Membranes were then probed using the indicated primary antibodies, all obtained from Cell Signaling Technology (Danvers, MA), at 1:1000 dilutions, except in the case of cytosolic loading control β-actin which was diluted 1:5000. IRDye 680LT goat anti-mouse and IRDye 800CW goat anti-rabbit secondary antibodies from Li-COR, Inc (Lincoln, NE) were used at 1:10,000 dilutions for visualization using the Li-COR Odyssey Classic Imaging System scanner. Images obtained using this scanner were analyzed with the Li-COR Image Studio Software version 5.2.5. Fluorescent signals were normalized to loading controls β-actin, or cytochrome C oxidase subunit IV (COX IV) for cytosolic and mitochondrial protein fractions, respectively. Average relative protein expressions of experimental treatment groups were determined by comparison to average expression of the control.

### Assay for measurement of reactive oxygen species production

HT22 cells were either untreated or treated for 24 h with 250 µM CoCl_2_, with and without rapamycin (500 nM), in 96 well plates with cells at around 70% confluence. 5 μM Dihydroethidium (DHE) (Invitrogen, Carlsbad, CA) in DMEM was added during the last 30 min of treatment time with incubation continuing at 37 °C. DHE is a cell permeable dye that becomes oxidized into a fluorescent compound, 2-hydroxyethidium, when the ROS indicator, superoxide, is produced in cells. Increased fluorescence, therefore, corresponds to increased ROS production. At the end of the 24 h treatment time, media was removed and cells were washed twice with PBS. A final volume of 100 µl PBS was added to each well prior to measuring fluorescence using a PHERAstar Microplate Reader with a 590-50/675-50 filter. Background fluorescence was subtracted using additional treatment sets without DHE. To compensate for fluorescence signal changes caused by cell death, resazurin cell viability assays, as described above, were performed in parallel using the same samples used to measure ROS production. Fluorescence measurements were normalized against cell viability to calculate the relative fluorescence values of control versus rapamycin-treated cells in which an increase in fluorescence is indicative of an increase in ROS production.

### Assay for measurement of the mitochondrial membrane potential (Δψ_m_)

Measurement of Δψ_m_ was performed essentially as previously described [31]. HT22 cells were cultured in 96 well plates to around 70% confluence. Cells were then either untreated or treated with 250 μM CoCl_2_, with and without rapamycin (500 nM) for 24 h at 37 °C with 500 nM tetramethylrhodamine, methyl ester (TMRM) (Thermo Fisher Scientific, Waltham, MA) being added during the final 30 min of treatment time. During the final 30 min of treatment time, 500 nM tetramethylrhodamine, methyl ester (TMRM) in DMEM medium was added to each well. At the end of treatment time, TMRM-containing media was removed and cells were washed twice with PBS. A final volume of 100 µL PBS was added to each well prior to reading fluorescence with a PHERAstar Microplate Reader (BMG Labtech, Durham, NC) using a 590-50/675-50 filter. Background fluorescence was subtracted using additional treatment sets without TMRM. To compensate for fluorescence changes caused by cell death, resazurin cell viability assays, as described above, were performed in parallel using the same samples used to measure the Δψ_m_. Relative TMRM fluorescence values were calculated by normalizing TMRM fluorescent measurements against cell viability measurements.

### Statistical analysis

Each experiment was repeated at least three times with each individual experiment using triplicate samples. Data are presented as either mean values ± standard deviation (SD), or as a percentage of the control. Statistical analysis was carried out using two-sided *t* tests, with p-values ≤ 0.05 considered statistically significant.

## Abbreviations

Δψ_m_: mitochondrial membrane potential
AIF: apoptosis inducing factor
ATP: adenosine triphosphate
Bax: Bcl-2 associated ×
Bcl-2: B cell lymphoma
CoCl_2_: cobalt chloride
COX IV: cytochrome C oxidase subunit IV
DHE: dihydroethidium
DMEM: Dulbecco’s modified eagles medium
DMSO: dimethyl sulfoxide
HIF-1α: hypoxia inducible factor-1alpha
IAPs: inhibitors of apoptosis
LC3: microtuble-associated protein light chain
MAPK: mitogen-activated protein kinase
MOMP: mitochondrial outer membrane permeability
mPTP: mitochondrial permeability transition pore
mTOR: mammalian target of rapamycin
pMAPK: phosphorylated mitogen-activated protein kinase
p-mTOR: phosphorylated mTOR
pS6: phospho-S6 ribosomal protein
ROS: reactive oxygen species
TBI: traumatic brain injury
TMRM: tetramethylrhodamine, methyl ester
